# Mitochondrial DNA in the tumour microenvironment activates neutrophils and is associated with worse outcomes in patients with advanced epithelial ovarian cancer

**DOI:** 10.1038/s41416-018-0339-8

**Published:** 2018-12-06

**Authors:** Kelly L. Singel, Kassondra S. Grzankowski, A. N. M. Nazmul H. Khan, Melissa J. Grimm, Anthony C. D’Auria, Kayla Morrell, Kevin H. Eng, Bonnie Hylander, Paul C. Mayor, Tiffany R. Emmons, Nikolett Lénárt, Rebeka Fekete, Zsuzsanna Környei, Uma Muthukrishnan, Jonathan D. Gilthorpe, Constantin F. Urban, Kiyoshi Itagaki, Carl J. Hauser, Cynthia Leifer, Kirsten B. Moysich, Kunle Odunsi, Ádám Dénes, Brahm H. Segal

**Affiliations:** 1 Department of Immunology, Roswell Park Comprehensive Cancer Center, Buffalo, NY USA; 2 Department of Surgery, Division of Gynecologic Oncology, Roswell Park Comprehensive Cancer Center, Buffalo, NY USA; 3 Arizona Center for Cancer Care, Phoenix, AZ USA; 4 Department of Medicine, Roswell Park Comprehensive Cancer Center, Buffalo, NY USA; 5 Department of Biostatistics and Bioinformatics, Roswell Park Comprehensive Cancer Center, Buffalo, NY USA; 6 Momentum Laboratory of Neuroimmunology, Institute of Experimental Medicine, Hungarian Academy of Sciences, Budapest, Hungary; 7 Department of Pharmacology and Clinical Neuroscience, Umeå University, Umeå, Sweden; 8 Department of Clinical Microbiology, Umeå University, Umeå, Sweden; 9 Department of Surgery, Beth Israel Deaconess Medical Center, Harvard Medical School, Boston, MA USA; 10 Department of Microbiology and Immunology, Cornell University College of Veterinary Medicine, Ithaca, NY USA; 11 Department of Cancer Prevention and Control, Roswell Park Comprehensive Cancer Center, Buffalo, NY USA; 12 Center for Immunotherapy, Roswell Park Comprehensive Cancer Center, Buffalo, NY USA; 13 Department of Medicine, Jacobs School of Medicine and Biomedical Sciences, University at Buffalo, Buffalo, NY USA

**Keywords:** Ovarian cancer, Tumour immunology, Tumour biomarkers, Coagulation system, Neutrophils

## Abstract

**Background:**

Advanced cancer causes necrosis and releases damage-associated molecular patterns (DAMPs). Mitochondrial DAMPs activate neutrophils, including generation of neutrophil extracellular traps (NETs), which are injurious, thrombogenic, and implicated in metastasis. We hypothesised that extracellular mitochondrial DNA (mtDNA) in ascites from patients with epithelial ovarian cancer (EOC) would correlate with worse outcomes.

**Methods:**

Banked ascites supernatants from patients with newly diagnosed advanced EOC were analysed for mtDNA, neutrophil elastase, and activation of healthy donor neutrophils and platelets. TCGA was mined for expression of *SELP* and *ELANE*.

**Results:**

The highest quartile of ascites mtDNA correlated with reduced progression-free survival (PFS) and a higher likelihood of disease progression within 12-months following primary surgery (*n* = 68, log-rank, *p* *=* *0.0178*). NETs were detected in resected tumours. Ascites supernatants chemoattracted neutrophils, induced NETs, and activated platelets. Ascites exposure rendered neutrophils suppressive, based on abrogation of ex vivo stimulated T cell proliferation. Increased *SELP* mRNA expression correlated with worse overall survival (*n* = 302, Cox model, *p* *=* *0.02*).

**Conclusion:**

In this single-centre retrospective analysis, ascites mtDNA correlated with worse PFS in advanced EOC. Mitochondrial and other DAMPs in ascites may activate neutrophil and platelet responses that facilitate metastasis and obstruct anti-tumour immunity. These pathways are potential prognostic markers and therapeutic targets.

## Background

Epithelial ovarian cancer (EOC) is the leading cause of death from gynaecological malignancies and the second most common gynaecological cancer in the United States. In an analysis of 1,895 patients with stage III EOC treated with primary surgery and standard chemotherapy, the median progression-free survival (PFS) was 17 months and the median overall survival (OS) was 45 months.^1^ Prognostic factors for advanced EOC include age, performance status, residual tumour volume, tumour histology, and serum CA-125 levels.^1,2^ The immune responses in the tumour microenvironment can also influence clinical outcome and are potential targets for therapeutic modulation. Tumour-infiltrating T cell accumulation and high CD8^+^ to regulatory T cell ratio predicted better outcomes, while increased regulatory T cell accumulation predicted worse outcomes in patients with advanced EOC.^3–5^ The tumour microenvironment of EOC is inflammatory as well as immunosuppressive, and characterised by the accumulation of ascites, and mature and immature myeloid cells, cytokines, and chemokines.^6–11^ Cui et al.^12^ reported that myeloid cell accumulation in EOC triggered acquisition of stem cell-like features in cancer cells, increased metastatic potential, and was also associated with worse outcomes. Although clinical variables such as tumour stage and histology cannot be modified, the immune responses shaping the tumour microenvironment are potential novel prognostic biomarkers and therapeutic targets.

Neutrophils and platelets become activated as emergency responders to infection and injury, and co-migrate to sites of injury.^13^ While initial responses are critical for defence against infection, control of bleeding and promotion of wound repair, these responses can accelerate tumour progression when active in the tumour microenvironment. Labelle et al.^14^ showed that platelet-derived chemokines CXCL5/7 guide granulocytes to circulating tumour cells. This platelet-granulocyte interaction with tumour cells promotes tumour seeding and metastasis. In patients with EOC, elevated pre-treatment circulating neutrophil counts^15^ and high neutrophil-to-lymphocyte ratio^16^ correlated with poor outcomes. Paraneoplastic thrombocytosis predicted worse outcomes in patients with a number of solid tumours, including newly diagnosed advanced EOC.^17,18^ In addition, platelet infiltration into tumours after anti-angiogenic therapy withdrawal led to enhanced tumour rebound.^19^

Mitochondrial DAMPs (mtDAMPs), which include mitochondrial DNA (mtDNA) and formylated peptides, are released following traumatic injury and activate neutrophils through ligation of toll-like receptor 9 (TLR9) and formylated peptide receptor. We previously observed that mtDAMPs stimulated the generation of neutrophil extracellular traps (NETs).^20^ NETosis is a distinct mode of neutrophil death characterised by the breakdown of membranes and extracellular release of chromatin and granular constituents.^21^ NETosis results in the release of products that can augment antimicrobial host defence but also cause tissue injury.^22^ NETs have been shown to promote thrombosis, likely through the release of extracellular chromatin^23–25^ and tissue factor,^26^ while P-selectin also promotes NETosis.^27^ In addition, NETosis is implicated in both cancer-associated thrombosis^24^ and acceleration of metastasis.^28,29^ Taken together, different experimental models point to injury and release of DAMPs in the tumour microenvironment stimulating neutrophilic and pro-thrombogenic responses that can enhance tumour progression. We hypothesised that mtDNA in the tumour microenvironment activates neutrophil and platelet wound repair responses and correlates with worse outcomes in patients with newly diagnosed advanced EOC.

Previously, we reported that the combination of high tumour necrosis factor-alpha and IL-6 in the ascites was associated with reduced PFS in patients with newly diagnosed advanced EOC.^30^ This signature suggested an interaction between proinflammatory pathways driving tumour progression and resistance to chemotherapy in advanced EOC. In addition, we observed that granulocytes isolated from the ascites suppressed stimulated T cell proliferation ex vivo.^31^ Here we investigated mtDNA in the ascites as a potential driver for pro-inflammatory pathways and reduced survival. Together, our results suggest that products of injury (e.g., mtDNA), and the subsequent neutrophil and platelet responses, are potential prognostic biomarkers and novel therapeutic targets in patients with advanced EOC.

## Methods

### Patients and Specimens

This study was approved by the Institutional Review Board (IRB) of Roswell Park Comprehensive Cancer Center (Roswell Park), Buffalo, NY, and was in compliance with federal and state requirements. All participants gave informed consent to use samples for research. Ascites was collected and processed from patients with newly diagnosed advanced EOC, as previously described.^30^ The medical records of these patients were retrospectively reviewed for PFS and OS calculated from time of diagnosis. Recurrence was defined by objective criteria based on CT scan imaging. PFS was the interval between diagnosis to disease progression, recurrence, or death. OS was the interval from diagnosis to date of death or censored at date of last follow-up. Banked ascites supernatants from patients with cirrhosis and without cancer were provided by Dr. Thomas Russo (Jacobs School of Medicine and Biomedical Sciences, University at Buffalo).

### Mice

Mice with a targeted disruption of the p47^*phox*^ gene (p47^*phox*−/−^) have a defective phagocyte NADPH oxidase (NOX2), rendering phagocytes incapable of generating measurable superoxide. p47^*phox*−/−^ mice were backcrossed 14 generations in the C57BL/6Ncr background. A p47^*phox*−/−^ mouse breeding colony is established at Roswell Park. C57BL/6Ncr mice were purchased from NCI. Bones from wildtype and B6.129P2-TLR9^tmAki^ (TLR9^−/−^) mice (backcrossed 11 generations in the C57BL/6 background) were obtained from Dr. Cynthia Leifer (Cornell University College of Veterinary Medicine, Ithaca, NY). Female mice (age 6–8 weeks) were used in all experiments. Animals were bred and maintained under specific pathogen-free conditions at the animal care facility at Roswell Park and used in compliance with all relevant laws and institutional guidelines under a protocol approved by the Institutional Animal Care and Use Committee.

### Purification of murine bone marrow neutrophils

Bone marrow-purified neutrophils (BM-PMNs) were isolated from 10-week-old female C57BL/6Ncr wildtype (WT) and p47^*phox*−/−^ mice. Briefly, long bones were harvested from each mouse and flushed with DPBS using a 26 G needle. Single-cell suspensions were generated and centrifuged on Percoll (#P1644, MilliporeSigma, St. Louis, MO, USA) density gradients to isolate BM-PMNs. The purity of BM-PMNs was >90% based on cytology.

### Activation of murine bone marrow-derived dendritic cells

Bone marrow was harvested from the long bones of WT and TLR9^−/−^ mice as described. To generate bone-marrow-derived dendritic cells (BMDCs), single-cell suspensions were generated and cultured in RPMI 1640 complete media containing murine GM-CSF (20 ng/ml) for 6–7 days. Media were refreshed every 2 days. For in vitro activation, BMDCs were treated with mtDNA (25 μg/ml) or CpG sequences (2 μM) for 24 h in media without GM-CSF. Polymixin B (10 µg/ml) was added with mtDNA as a specificity control. Flow cytometry analysis was conducted on a BD LSR II FACScan (Becton, Dickinson and Company, Franklin Lakes, NJ, USA). Forward scatter versus side scatter gating was set to include all non-aggregated cells from at least 20,000 events collected per sample.

### Isolation of donor neutrophils

Peripheral blood was collected from healthy donors after receiving written informed consent under an IRB-approved protocol. Neutrophils were isolated from peripheral blood by 2-step density gradient separation. First, blood was centrifuged on Histopaque (#1119, MilliporeSigma) to separate the granulocyte-rich fraction. Then neutrophils were isolated from the granulocyte-rich fraction by a second centrifugation on Percoll density gradients. The purity of neutrophils was >90% based on cytology and CD33^+^CD15^+^ expression.

### Extraction of mitochondrial DAMPs, mitochondrial DNA, and genomic DNA

De-identified remnant benign margins of resected liver from patients undergoing surgery for hepatic tumours were used as an abundant source of genomic DNA (gDNA) and mtDNA under an IRB-approved protocol. Mitochondrial DAMPs were isolated as previously described.^32^ Briefly, 200 mg of liver was sonicated on ice at 100% amplitude (30-sec each time with 30-s intervals, 10 times). The disrupted mitochondrial suspensions were centrifuged at 12,000 g for 10 min and at 10,000 g for 30 min. Supernatants were used in experiments as mtDAMPs; the yield of mtDAMPs is similar to that recovered *in vivo* in experimental 5% liver injury models. mtDNA was extracted using the mtDNA Extractor CT kit (#291-55301, Wako Chemicals, Richmond, VA, USA) following the manufacturer’s protocol. gDNA was extracted using QIAmp DNA Mini Kit following the manufacturer’s protocol (#51304, Qiagen, Germantown, MD, USA).

### Quantification of mitochondrial DNA and genomic DNA

Total DNA was extracted from ascites supernatants using the DNA purification kit (#51106, Qiagen) following the manufacturer’s protocol. Purified mtDNA from human liver, SYBR Green ER SuperMix kit (#11760, Thermo Fisher Scientific, Waltham, MA, USA), and mitochondrial-specific human cytochrome B primers (forward 5ʹ-ATGACCCCAATACGCAAAAT-3ʹ and reverse 5ʹ-CGAAGTTTCATCATGCGGAG-3ʹ) for real-time quantitative PCR (qPCR) were used to evaluate mtDNA in total DNA isolated from ascites.^32^ A similar method was applied to evaluate gDNA in ascites. qPCR analysis was conducted on the ABI Fast Real-Time instrument (Thermo Fisher Scientific).

### Detection of neutrophil extracellular traps ex vivo

BM-PMNs purified from WT and p47^*phox*−/−^ mice and circulating neutrophils from healthy donors were evaluated for NETosis. NETs were detected by immunofluorescent confocal microscopy using previously published methods.^33^ Neutrophils were exposed to phorbol 12-myristate 13-acetate (PMA, 100 nM) as a positive control, mtDAMPs, or ascites supernatants for 1 h. NETs were identified by immunofluorescent confocal microscopy based on extracellular stretches of DNA, as previously described.^33^ Immunostaining and confocal microscopy were performed as described in supplementary information. The major endpoints evaluated were the presence of NETs (yes/no) or NE levels in supernatants by ELISA.

### Immunostaining of resected EOC from patients

NETs were assessed in resected tumour from patients who underwent primary surgery for advanced EOC at Roswell Park. 5 µm FFPE sections were deparaffinised, rehydrated in decreasing concentrations of ethanol, and subjected to antigen retrieval by heating in 10 mM citrate buffer (pH 6.0) for 10 min. Specimens were blocked with a solution of 2% bovine serum albumin and 5% donkey serum in PBS + 0.1% Triton X-100 for 1 h at room temperature. Immunostaining and confocal microscopy were performed as described in supplementary information.

### Evaluation of neutrophil chemotaxis in response to Ascites in vitro

A 96-well plate system with top and bottom chambers separated by a transmembrane with 3 μm pores was used. Neutrophils from healthy donors (10^5^ cells/well) were plated in the top chamber. To attract the neutrophils, 5% mtDAMPs prepared from human liver or 10% ascites supernatants were added to the bottom chamber and incubated for 1 h at 37°C in 5% CO_2_. Neutrophils were pre-treated with 0.1% DMSO or 3 µM sphingosine kinase inhibitor (SKI). Adherent cells were detached from the transmembrane with lysis buffer and combined with the neutrophils that had migrated into the bottom chamber. Neutrophils were labelled with CyQuant GR dye (#C7026, Thermo Fisher Scientific) and evaluated against a standard curve to determine number of migrated cells.

### Identification of platelet microparticles by flow cytometry

To isolate and prepare platelet microparticles (PMPs), ascites supernatants were sequentially centrifuged at 500 × *g* for 10 minutes, 2000g for 10 minutes, and 25,000 × *g* for 20 minutes. PMPs ranging from 500 to 1000 nm were observed in the 25,000 × *g* pellet. Samples were stained with anti-CD41a (#12-0419, Thermo Fisher Scientific) and visualised on a BD Fortessa flow cytometer (Becton, Dickinson and Company).

### Isolation of platelets from donor and murine peripheral blood

Platelets were isolated from healthy donor peripheral blood or blood from the right cardiac ventricle of anesthetised male C57BL/6 mice (breeding at the Institute of Experimental Medicine, Budapest, Hungary). Blood was anticoagulated with 10% volume of acid citrate dextrose (ACD). Methods are described in more detail in supplementary information.

### Statistical analysis

All statistical analyses were performed using the R 3.4.0 statistical computing language. A nominal significance threshold of 0.05 was used unless otherwise specified. Statistical testing included Student’s *t* test, *χ*^2^ and Fisher’s exact tests, and Kaplan–Meier survival analysis with log-rank testing. The multivariate analysis comprised FIGO stage, categorised as early (I, II, or IIIA/B) or late (IIIC or IV), histological grade, debulking status (optimal defined by 1 cm margin and R0 defined as no macroscopic residual disease), and platinum-sensitive versus refractory disease. Patients were censored from the multivariate analysis if they were reported as alive with no evidence of disease. Restricted mean survival (RMS) curves described by Eng et al.^34,35^ were computed using ascites NE levels as a continuous variable.

TCGA provisional RNA Seq V2 RSEM data were downloaded from cBioportal for ovarian serous cystadenocarcinoma and mined for *ELANE*, *SELP*, and *ITGB3* expression. OS (*n* = 302) data was analysed for each primary tumour sample from cBioportal. Using Cox-Proportional Hazards Regression model, RMS curves were created using quantiles of the genes.

## Results

### High ascites mtDNA and neutrophil elastase is associated with reduced progression-free survival following primary surgery

**Fig. 1:**
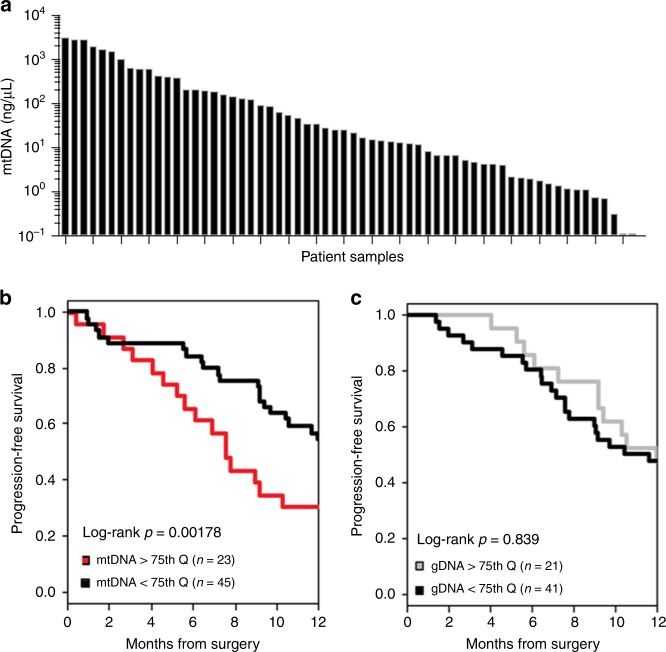
High ascites mtDNA levels are associated with reduced progression-free survival following primary surgery. **a**–**c** High ascites levels of mtDNA, but not gDNA, are associated with reduced median PFS. Banked ascites supernatants from patients with newly diagnosed advanced EOC were analysed for mtDNA and gDNA. **a** Variability exists between ascites mtDNA (ng/μL) levels as measured by qPCR (*n* = 68). **b** High mtDNA levels as defined by the upper 75^th^ quartile (red line) are associated with reduced PFS as compared to the lowest 75^th^ quartile (black line) of mtDNA (*n* = 68, log-rank, *p* = 0.0178). **c** Ascites gDNA levels are not associated with PFS (*n* = 62, log-rank, *p* = 0.839)

Banked ascites supernatants (*n* = 80) from patients with newly diagnosed EOC were analysed for mtDNA. Twelve patients were excluded based on early stage (I or II) or non-EOC histology. We observed substantial variability in the mtDNA concentration in ascites supernatants (*n* = 68; mean 298 ng/μL, range < 1 to 3139 ng/μL) **(**Fig. 1a**)**. We found that levels of mtDNA (log-rank, *p* = 0.0178, Fig. 1b), but not genomic DNA (gDNA, log-rank, *p* = 0.839, Fig. 1c), were associated with reduced median PFS. This effect was observed over a range of mtDNA percentiles (top tertile, quartile, and quintile). The mtDNA^high^ group was specified as patients with ascites mtDNA >75th quartile, and the mtDNA^low^ group was specified as patients with ascites mtDNA < 75^th^ quartile (Table 1). The 12-month PFS was the primary clinical endpoint. 23/68 (34%) patients were in the mtDNA^high^ group. Twenty-six percent (26%) of patients in the mtDNA^high^ and 16% in the mtDNA^low^ sub-groups were Stage IV. The mean age at diagnosis was 66.0 years in the mtDNA^high^ group and 60.4 years in the mtDNA^low^ group. Histology and grade were not significantly different between the two groups with majority being high-grade (3) serous ovarian cancer (HGSOC; accounts for 70% of EOC cases). In addition, we censored 7 patients based on their clinical status reported as alive with no evidence of disease, thus 61/80 patients were included in the multivariate analysis. Using the Cox model, the median PFS in the mtDNA^high^ and mtDNA^low^ groups was 7.6 (HR = 1.86, 95% CI: 5.61-15.80) vs. 15.2 (HR = 0.54, 95% CI: 10.43-19.00) months, respectively (*p* = 0.26).

**Table 1:** Ascites mtDNA levels stratified into lowest and highest 75^th^ quartiles are associated with reduced 12-month survival in patients with newly diagnosed advanced EOC

	mtDNA level		
	<75th Q	>75th Q	*p* value
*N*	45	23	
Age, Mean	60.4	66.0	0.06
Primary Site			0.26
Ovary	80%	70%	
Fallopian Tube	0%	4%	
Primary Peritoneal	20%	26%	
Stage			0.19
IIIB	0%	4%	
IIIC	84%	70%	
IV	16%	26%	
Grade			0.76
Well Differentiated	5%	5%	
Moderately Differentiated	14%	23%	
Poorly Differentiated	81%	73%	
Histology			0.30
Serous	82%	70%	
Clear Cell	4%	0%	
Endometrioid	0%	4%	
Mucinous	2%	4%	
Mixed	11%	22%	
Debulking			0.73
Optimal	71%	78%	
Sub-optimal	29%	22%	
Residual Tumour			
R0^a^	2%	9%	0.26
Not R0	98%	91%	
Platinum Status			0.23
Sensitive	58%	39%	
Refractory/Resistant	42%	61%	
Progression-free survival			
Events	40	21	
Median PFS [95% CI]	15.18 [10.43−19.00]	7.57 [5.61−15.80]	0.26
PFS to 12 months (std. error)	0.55 (0.08)	0.30 (0.10)	0.002
PFS to 24 months (std. error)	0.22 (0.06)	0.17 (0.08)	0.28
HR (PFS)	0.73	1.36	0.26
Adj HR^b^	0.54	1.86	0.04

^a^R0: no macroscopic residual disease

^b^Adjusted for age, stage, grade, debulking status

Since mtDAMPs activate neutrophils, including degranulation^32^ and generation of NETs,^20^ we evaluated two products of neutrophil degranulation, neutrophil elastase (NE) and myeloperoxidase, in banked ascites supernatants. Patients with the highest levels of ascites NE had a significantly lower likelihood of achieving PFS at 12 months (*n* = 67, log-rank, *p* < 0.001) (Table 2), while myeloperoxidase levels did not correlate with outcome. Considering the ascites NE levels as a continuous variable, we evaluated the restricted mean survival (RMS) curve (Supplemental Fig. 1). The RMS curve shows that with higher levels of ascites NE (e.g., 1200 ng/mL), patients had a survival of approximately 8.5 months, whereas with lower levels of ascites NE (e.g., 200 ng/mL), patients survived approximately 11.5 months. However, these are the extremes of ascites NE levels, and the relationship was not linear. The RMS curve highlights the complexity of the relationship between ascites NE levels and survival. Together, these results point to ascites mtDNA and NE levels at diagnosis of advanced EOC as potential prognostic biomarkers.

**Table 2:** Ascites neutrophil elastase stratified into low, intermediate, and high levels predicts reduced progression-free survival within 12 months following surgery

	Neutrophil Elastase Level			
	<377 ng/mL	377 < ng/mL <1222	>1122 ng/mL	*p* value
*N*	11	52	10	
Age, Mean	65.3	61.4	62.7	ns
Stage (% IV)	9%	23%	10%	ns
Grade (% 3)	81%	76%	21%	ns
Debulking (% Optimal)	36%	23%	13%	ns
Progression-free survival				
Events	9	49	9	
Median PFS [95% CI]	16.26 [5.88-NA]	9.56 [6.51-15.0]	5.57 [0.29-NA]	0.0328
PFS to 12 months (std. error)	0.70 (0.144)	0.42 (0.069)	0.20 (0.127)	< 0.001
PFS to 24 months (std. error)	0.12 (0.109)	0.10 (0.044)	0.10 (0.095)	ns
HR (PFS)	0.41	1	2.05	0.0367
Adj HR^a^	0.36	1	2.94	0.039

^a^Adjusted for Age, stage, grade, debulking status

### Ovarian cancer ascites is chemoattractive to neutrophils and induces neutrophil extracellular traps and suppressive neutrophils

**Fig. 2:**
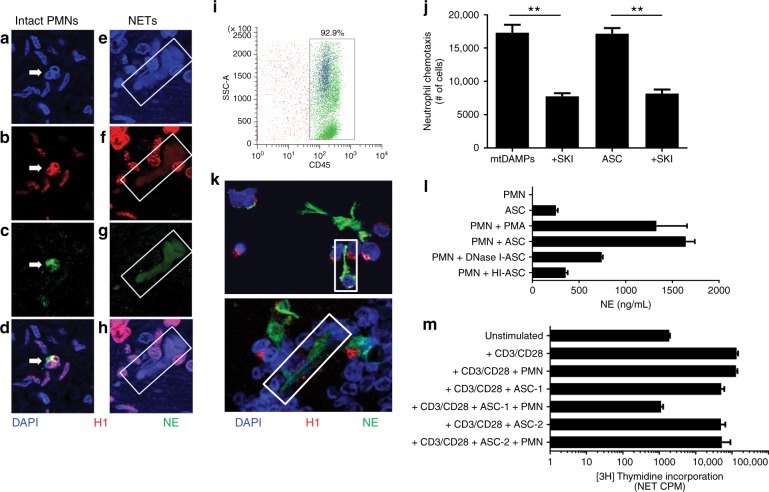
Ovarian cancer ascites is chemoattractive to neutrophils and induces NETs and suppressive neutrophils. **a**–**h** Resected tumours were collected from patients with newly diagnosed advanced EOC (*n* = 5). Tumours were evaluated for the presence of (**a**–**d**) intact neutrophils (PMNs, white arrows), identified by a hypersegmented nucleus (DNA, blue; histone H1, red), and cytoplasmic NE (green), and (**e**–**h**) NETs, identified by co-localised extracellular DNA, histone H1, and NE (white boxes). PMNs within tumours were rare. NETs were visualised in 4/5 tumours evaluated. **i** Ascites were collected from patients with newly diagnosed HGSOC (*n* = 9). >90% of the cells are CD45^+^ leukocytes and on average 15–20% are granulocytic (blue). **j**–**m** Ascites supernatants (ASC) were evaluated for pathways of PMN activation. PMNs were isolated from peripheral blood of healthy donors. **j** Donor PMN chemotaxis was measured in response to mtDAMPs (positive control) and ascites supernatants (*n* = 2). Sphingosine kinase inhibitor (SKI) was added to both mtDAMPs and ascites supernatants as a negative control to inhibit chemotaxis (**p *<* *0.01*). **k** Donor PMNs were treated for 1 h with ascites supernatants and evaluated for NET generation (white boxes) by immunofluorescent confocal microscopy. Exposure of neutrophils to 3 of 4 ascites supernatants resulted in NETs. **l** Donor PMNs were treated for 1 h with media, 100 nM PMA (positive control), ascites supernatants (*n* = 4), DNase I-pre-treated ascites supernatants, or heat-inactivated ascites supernatants, and evaluated for degranulation by NE ELISA. Ascites-induced neutrophil degranulation, which was partially reversed with heat-inactivation (HI-ASC) or DNase I pre-treatment. **m** Autologous donor PMNs, CD4^+^, and CD8^+^ T cells were isolated and used in co-culture. Neither PMNs nor ascites supernatants alone suppress anti-CD3/CD28-stimulated T cell proliferation, however, the majority of ascites supernatants from patients with newly diagnosed HGSOC (*n* = 17/22) induced PMNs to suppress T cell proliferation. ASC-1 is an example of an ascites sample that induced the neutrophil suppressor phenotype, and ASC-2 is an example of an ascites sample that did not induce the neutrophil suppressor phenotype. Data are from ≥3 independent experiments

Since NETs have been visualised in solid tumours,^29,36,37^ we determined whether NETosis occurs in tumour-infiltrating neutrophils at primary surgery for EOC. Tumour-infiltrating neutrophils were uncommonly observed, but both intact neutrophils (Fig. 2a–d) and NETs (Fig. 2e-h) were observed in tumours from 4/5 patients.

In ascites from patients with newly diagnosed advanced EOC, the predominant cellular population was leukocytes (CD45^+^) (mean 92.9%, *n* = 9 HGSOC) (Fig. 2i). As we previously reported, CD15^+^ neutrophils were ~15–20% of ascites cells.^31^ Since neutrophils are short-lived, ongoing neutrophil recruitment is required to maintain neutrophilic inflammation. Therefore, we evaluated whether ascites supernatants act as a chemoattractant for neutrophils. Using mtDAMPs as a positive control, we found that ascites supernatants stimulated neutrophil chemotaxis (Fig. 2j), and that sphingosine kinase inhibitor (SKI), which inhibits calcium influx, reduced chemotaxis elicited by both mtDAMPs and ascites supernatants.

Next, we evaluated whether ascites supernatants stimulated NETosis. NETosis can occur through NADPH oxidase (NOX2)-dependent^33^ and -independent pathways.^38^ First, we asked whether mtDAMPs stimulated NOX2 and if NETosis was NOX2-dependent. Using purified BM-PMNs from WT and NOX2-deficient p47^*phox-/-*^ mice, we found that mtDAMPs activated NOX2 as measured by DHR123 fluorescence (Supplemental Fig. 2A). PMA was used as a positive control for NOX2-dependent NETosis,^39^ and, as expected, PMA did not induce NETs in p47^*phox−/−*^ BM-PMNs (Supplemental Fig. 2B, left upper panel). In contrast, mtDAMPs stimulated NETosis in p47^*phox−/−*^ BM-PMNs, demonstrating NOX2-independent NETosis (Supplemental Fig. 2B, right upper panel). Consistent with mouse BM-PMNs and our prior study,^20^ PMA and mtDAMPs induced NETs in neutrophils from healthy donors (Supplemental Fig. 2B, lower panels). Exposure of neutrophils to 3 of 4 ascites supernatants resulted in NETs as visualised by immunofluorescent confocal microscopy (Fig. 2k). Consistent with induction of NETosis, exposure of neutrophils to ascites supernatants led to NE release **(**Fig. 2l**)**. Heat-inactivating ascites supernatants before neutrophil exposure reduced the amount of NE released into supernatants (Fig. 2l). Pre-treating ascites supernatants with DNase I to deplete both gDNA and mtDNA also reduced NE release, but to a lesser extent than heat inactivation (Fig. 2l). Thus, mtDAMPs and ascites supernatants induce NETosis through NOX2-independent pathways, and release of NE is abrogated by prior treatment of ascites supernatants with heat and DNase I.

Finally, since we previously observed that purified ascites granulocytes suppressed stimulated T-cell proliferation,^31^ we asked whether ascites supernatants would induce a similar suppressor phenotype in circulating neutrophils from healthy donors. In a separate cohort of patients, we found that 17 of 22 HGSOC ascites supernatants induced neutrophils to acquire a suppressor phenotype, abrogating ex vivo anti-CD3/CD28-stimulated T cell proliferation (Fig. 2m). Together these results point to ascites inducing neutrophil recruitment and activation, as well as a suppressor phenotype in the tumour microenvironment.

### Mitochondrial DAMPs activate DCs through TLR9-dependent and -independent pathways

Zhang et al.^32^ showed that mtDAMPs activated neutrophils through both TLR9 and formylated peptide receptor. We previously showed that immunisation with an adjuvant containing TLR9 and NOD2 ligands significantly prolonged survival in EOC tumour-bearing mice.^31^ Therefore, we asked whether mtDNA activated DCs and whether activation was TLR9-dependent. WT and TLR9^−/−^ BMDCs were stimulated with mtDNA and CpG sequences, and surface markers for DC activation were assessed. While DC activation by CpG was TLR9-dependent, mtDNA-induced DC activation was variably TLR9-dependent (Supplemental Fig. 3). The mtDNA-induced upregulation of MHC class I and II and CD80 was TLR9-dependent, while upregulation of CD86 was TLR9-independent. Addition of polymyxin B did not alter the expression of costimulatory molecules, arguing against an effect by LPS contamination. These results suggest that mtDAMPs in the tumour microenvironment have broad effects on innate immunity with the potential to suppress or enhance anti-tumour immunity.

### Ovarian cancer ascites induces rapid platelet activation and aggregation

**Fig. 3:**
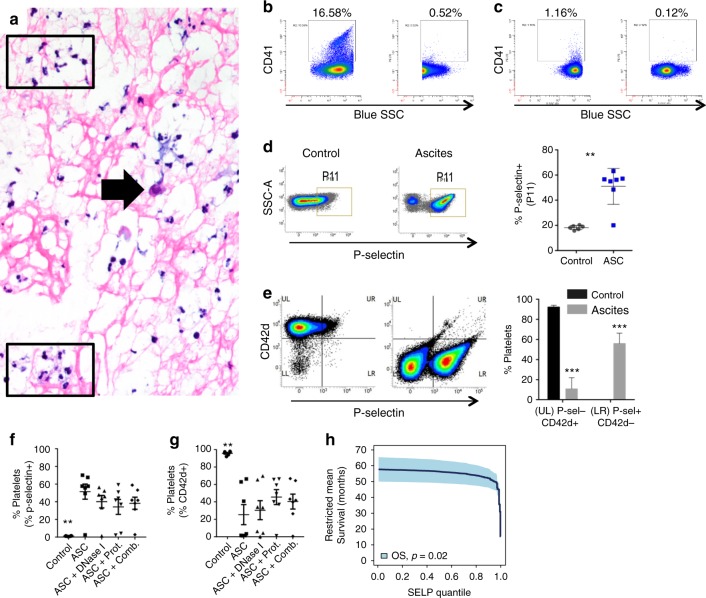
Ovarian cancer ascites induces rapid platelet activation and aggregation that is partially abrogated by DNase and protease treatments. **a**–**g** Ascites were collected from patients with newly diagnosed advanced EOC and 500 g supernatants (ASC) were used. **a** Floating aggregates were collected from ascites before centrifugation and analysed by H&E. Abundant neutrophils (black box) and a sparse number of tumour cells (black arrow) embedded in fibrin deposits (pink filaments) were identified. **b**, **c** CD41^+^ PMP were measured in ascites supernatants by modified flow cytometry (*n* = 2). Variability exists between different patients (B, left) 16.58% and (C, left) 1.16% CD41a^+^. In parallel, samples were 0.1 µm-filtered to remove microparticles as a specificity control (**b**, **c**, right panels). **d**–**g**. Platelets were isolated from peripheral blood of healthy donors and murine cardiac puncture. **d** Donor platelets were exposed to Tyrode’s buffer with 1 mM CaCl_2_ (negative control) or ascites supernatants (*n* = 7) in the presence of 1 mM CaCl_2_ for 30 minutes prior to staining for flow cytometry. Representative density plots show increased number of P-selectin^+^ platelets (P11 gate) after exposure to ascites supernatants, which is quantified to the right (**, *p* *<* *0.01*). E) Naïve murine platelets were exposed to the same ascites supernatants (*n* = 7), resulting in increased P-selectin expression and loss of CD42d from the surface of platelets within 15 minutes. The proportion of platelets in the upper left (UL; P-selectin^-^CD42d^+^; resting) and lower right (LR; P-selectin^+^CD42d^−^; activated) quadrants of the density plots are quantified to the right (***p *<* *0.001*). **f**, **g**) After treatment with ascites supernatants with or without DNase I (0.05% w/v) and/or protease inhibitors (1:100), the proportions of F) P-selectin^+^ donor platelets and (**g**) CD42d^+^ murine platelets are quantified. Data are from ≥ 3 independent experiments. **h** High P-selectin mRNA expression in tumour is associated with reduced OS following primary surgery. TCGA provisional RNA Seq V2 RSEM data from cBioportal for ovarian serous cystadenocarcinoma (*n* = 302) were mined for expression of *SELP*. Quantiles of *SELP* expression were plotted against restricted mean survival (RMS) and *SELP* expression was associated with worse OS (Cox model, *p* = 0.02, HR = 1.14, 95% CI: 1.03–1.28)

In unmanipulated ascites collected at primary surgery for EOC, we observed fibrin deposits with varying proportions of embedded tumour cells and neutrophils (Fig. 3a). The presence of neutrophils is indicative of active inflammation as opposed to clotting that can occur as a processing artifact. Platelet concentration in ascites was very low in relation to circulation, possibly because activated platelets rapidly degranulate. Platelets release microparticles during inflammation, which are internalised by neutrophils and amplify neutrophilic inflammation.^40,41^ We identified CD41a^+^ platelet microparticles (PMPs) in ascites supernatants with substantial variability between samples (Fig. 3b, c). Next, we evaluated mechanisms for ascites-induced platelet activation. Platelets from healthy donors were stimulated with ascites supernatants ex vivo (Supplemental Table 1). Ascites supernatants induced robust platelet activation and aggregation as indicated by an over three-fold increase in P-selectin-positive platelets within 30 minutes after stimulation with 6/7 ascites samples (Fig. 3d). Since healthy donors showed some variation in platelet P-selectin levels, we also tested the ability of ascites supernatants to induce activation of platelets isolated from naïve WT mice. Exposure to ascites supernatants induced a profound increase in P-selectin levels and a reduction in CD42d levels in mouse platelets within 15 minutes, confirming the effect seen with donor platelets (Fig. 3e). In addition, cirrhotic ascites supernatants phenocopied the effect seen with EOC ascites supernatants (Supplemental Fig. 4), suggesting that ascites, regardless of disease origin, activates platelets. These results support the notion that platelets are prone to activation outside of the circulation, particularly in response to an acute injury or inflammatory stimuli, and their responses are not specific to tumour-associated factors.

To investigate potential mechanisms for the observed pro-thrombogenic activity, EOC ascites supernatants were pre-treated with DNase I and/or protease inhibitors for 30 minutes prior to platelet stimulation. Protease inhibitors and DNase I had modest effects on P-selectin upregulation on donor platelets (Fig. 3f) with substantial inter-donor variability, and CD42d downregulation on murine platelets (Fig. 3g). Using murine platelets, we found that protease inhibitors were modestly more effective at preventing platelet activation as induced by ascites supernatants compared to DNase I. This suggests that there are multiple, most likely redundant, pathways through which ascites can be pro-thrombogenic and induce platelet activation.

Given the cross signalling between activated neutrophils and platelets, we mined TCGA data for mRNA expression of *ELANE*, *SELP*, and *ITGB3*, which encode NE, P-selectin, and integrin beta-3, respectively. NE is expressed by neutrophils, P-selectin can be expressed by both platelets and endothelial cells, and integrin beta-3 is expressed by platelets only. Analysing the *SELP* RMS curve for HGSOC (*n* = 302) resulted in a statistically significant reduction in OS (Cox model, *p* = 0.02, HR = 1.14, 95% CI: 1.03–1.28) **(**Fig. 3h**)**. Neither analyses for *ELANE* nor *ITGB3* varied significantly from a constant function. Together, these results show that EOC ascites is highly thrombogenic, and intratumoural P-selectin expression correlates with worse prognosis.

### Extracellular histones are present in ovarian cancer ascites with a predominance of H1

DNA is packed around core histones (e.g., H2A/B, H3, H4) to form the nucleosome, and linker histone H1 family members bind to nucleosomes to stabilise higher-order chromatin structure. Extracellular histones are released during NETosis and cellular injury and are thrombogenic. We therefore evaluated the histone composition in ascites and its effects on platelet activation. H1 and H3 were observed in both untreated ascites supernatants (500 g) and supernatants from ascites centrifuged at 10,000 × *g* (Supplemental Figure 5A-G). H1 was present in all ascites supernatants tested (*n* = 9) and was not depleted by centrifugation. This observation indicates that H1 was not associated with membranes and other particles that sediment with centrifugation. Conversely, H3 was only detected in 4/9 of the ascites supernatants and, with the exception of one sample, was not depleted by centrifugation. The concentration of H1 was 10-fold higher than the concentration of H3 in ascites supernatants. The mean nucleosome enrichment factor in ascites supernatants was similar to normal serum, though substantial interpatient variability in ascites supernatants was observed. Four of nine (4/9) ascites supernatants had enriched nucleosomes over normal serum levels (Supplemental Figure 5H). There were no significant differences between nucleosome enrichment in EOC patient sera versus normal serum.

Finally, we compared the effects of the ascites supernatants, 10,000 × *g* supernatants, and 10,000 × *g* pellets on platelet activation. All treatments significantly upregulated P-selectin on donor platelets (Supplemental Figure 6). In murine platelets, the 10,000 × *g* supernatants induced upregulation of P-selectin (2/4 ascites), whereas none of the 10,000 × *g* pellets caused upregulation of P-selectin (Supplemental Figure 7). Since we could not achieve complete depletion of histones from ascites, we cannot make conclusions about whether histones are required for ascites-induced platelet activation. The ability of soluble and sedimented ascites fractions to activate platelets suggests a number of redundant pro-thrombogenic pathways in ascites.

## Discussion

We previously observed that the volume of ascites at initial diagnosis of EOC correlated with worse PFS and OS, and that intraperitoneal administrations of murine ascites supernatants accelerated EOC progression in vivo.^42^ These results pointed to ascites driving tumour progression. Our results presented here point to mtDNA, and the subsequent neutrophil and platelet responses, as potential prognostic biomarkers and novel therapeutic targets in patients with advanced EOC. In patients with newly diagnosed advanced EOC treated at Roswell Park, the highest quartile of ascites mtDNA was associated with significantly shorter median PFS and a higher likelihood of disease progression within 12-months following primary surgery. Our studies focused on mtDNA and did not address the prognostic value of other DAMPs. Patients with the highest ascites NE levels had significantly shorter median PFS and a higher likelihood of disease progression within 12-months following primary surgery, but the relationship between NE levels and PFS was not linear. We measured NE in banked ascites supernatants by ELISA, which is not a direct measure of NETosis, but a marker of released neutrophil granular products that can occur through NETosis, degranulation, and neutrophil death. Importantly, the mtDNA and NE signatures were significant when restricted to a 12-month window after surgery—an interval that corresponds to clinically defined chemotherapy-refractory disease. This database is from a retrospective analysis from a single center and should therefore be considered exploratory. A larger study of prospectively enrolled patients with newly diagnosed advanced EOC is underway and will test validation of these findings.

TCGA analysis showed that increased mRNA expression of *SELP* in HGSOC resected at primary surgery associated with worse OS. Prior studies have shown that ascites is pro-thrombogenic and that thrombin can facilitate tumour invasion through modulation of macrophage function.^43,44^ In addition, Labelle et al.^14^ showed that granulocyte-platelet interactions promoted early metastatic niche that facilitated metastatic seeding and progression. This study focused on the early events promoting metastasis in an *in vivo* murine model, while the tumour microenvironment in EOC is characterised by persistent inflammation and injury. We observed fibrin aggregates, taken directly from the tumour microenvironment at surgery, embedded with neutrophils and tumour cells. Since fibrin adheres to surfaces, these fibrin-neutrophil-tumour cell networks may be important for seeding of serosal surfaces, a characteristic feature of advanced EOC. Platelets can also increase the proliferation of EOC cells through TGF-β-dependent signalling.^45^ Taken together, these results suggest that therapeutic targeting of neutrophil-platelet interactions may inhibit EOC progression and metastasis.

One mechanism by which neutrophils might accelerate tumour progression is through cross signalling with platelets. Platelet-derived mtDNA and microparticles in EOC ascites may be a mechanism for cross-activation of neutrophils and NETosis. This idea is supported indirectly by our observation that ascites levels of mtDNA (but not gDNA since platelets are anucleate) correlated with worse prognosis. An important observation was the substantial variability observed in the ability of DNase I and protease inhibitors to prevent platelet activation by ascites supernatants, suggesting that multiple pathways exist for platelet activation in the tumour microenvironment. Similar to EOC ascites, ascites supernatants from patients with cirrhosis also activated platelets, supporting the notion that platelets are prone to activation outside of the circulation, particularly in response to an acute injury or inflammatory stimuli, and their responses are not specific to tumour-associated factors. EOC ascites is highly inflammatory and injurious and contains fibrin deposits harbouring neutrophils and tumour cells, while cirrhotic ascites is typically transudative with few inflammatory cells. Cirrhotic ascites contains several procoagulants, including but not limited to pre-activated Factor X (Xa) and tissue factor.^46^ Tissue factor is also abundant in malignant pleural effusions from lung adenocarcinoma patients.^47^ Therefore, it is plausible that several procoagulants are present in all ascites, regardless of disease origin, and have the potential to activate platelets and thrombosis. Thus, in EOC, our data and results from others^14,19^ suggest that platelets are recruited to and activated in the tumour microenvironment where they drive thrombosis and metastasis. In addition, extracellular histones, which can result from NETosis, are highly injurious^48^ and can promote thrombosis.^25^ Histone H1 concentration was 10-fold higher in ascites supernatants than H3, which may be due to NETs releasing extracellular H1. A limitation of these studies is that we cannot distinguish the source of extracellular histones, whether from neutrophils or other cells.

**Fig. 4:**
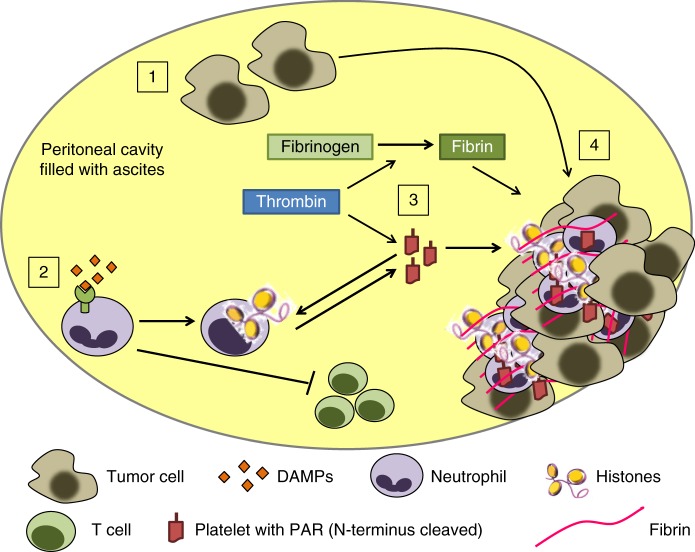
Model of DAMPs and neutrophil-platelet responses in the ascites of patients with advanced EOC. (1) A hallmark of advanced cancer is cellular necrosis, which releases DAMPs, and minor numbers of tumour cells into the ascites. (2) mtDNA, and likely other DAMPs, recruit and activate neutrophils, induce NETosis, and (3) activate platelets. (4) Platelet activation and aggregation with NETs and fibrin filaments trapfree floating tumour cells and enhance seeding to the serosa and local dissemination within the peritoneal cavity. In addition, neutrophils acquire a T cell suppressor phenotype after ascites exposure, an effect that is predicted to impair T cell immunity and obstruct immunotherapy

Considering the body of literature and our results on neutrophil-platelet interactions promoting tumour progression, we propose a model of EOC progression that links injury, neutrophilic inflammation, and thrombosis (Fig. 4). Cellular necrosis in the tumour microenvironment releases DAMPs (e.g., mtDNA), which recruit neutrophils. mtDNA within ascites stimulates NETosis, resulting in the release of proteases (e.g., NE and matrix metalloproteinases) that remodel extracellular matrix, and chromatin and tissue factor that promote platelet activation and thrombosis. While these responses serve to target microbes and prevent their dissemination, in the tumour microenvironment we posit that they enable tumour seeding of serosal surfaces where early metastasis in EOC occurs. In addition to the direct adhesive effect of fibrin, activated platelets cross signal with neutrophils and release pro-proliferative and angiogenic factors that enhance tumour progression and metastasis. In addition, ascites chemoattracts and induces a suppressor phenotype in neutrophils that is expected to be a barrier to tumour immunity. While strengths of our experimental approach include the use of primary cells and ascites as an authentic component of the EOC microenvironment, a limitation is that biological studies are ex vivo and don’t encapsulate the complexity of the tumour microenvironment.

Our model includes a number of potentially targetable pathways involving neutrophil recruitment and activation, including NETosis, thrombosis, and impairment of T cell-dependent anti-tumour immunity. Inhibition of neutrophil recruitment (e.g., with small molecule CXCR2 inhibitors) and NETosis has shown benefit in murine tumour models.^28,29,49^ Coffelt et al.^50^ showed in a mammary tumour model that tumour-induced neutrophils suppressed CTL responses, and depletion of IL-17 or G-CSF abrogated the T-cell suppressive phenotype of neutrophils. In addition, the absence of γδ T cells and neutrophils limited metastasis. McGray et al.^51^ recently showed that anti-Gr1 depletion enhanced the benefit of vaccination and PD-1 blockade in murine EOC. Here, we also observed that mtDNA activates DCs through TLR9-dependent and –independent pathways, an effect that may promote T cell immunity. Thus, mtDNA and other DAMPs can have multiple effects on innate immune responses that can either accelerate or inhibit tumour progression.

We previously observed that granulocytes isolated from the ascites suppressed stimulated T cell proliferation ex vivo.^31^ In the current study, ascites supernatants induced a suppressor phenotype in neutrophils from healthy donors, characterised by the abrogation of proliferation of ex vivo stimulated autologous T cells. It is well recognised that tumour-derived factors can induce a myeloid cell expansion in the marrow that leads to the generation of immature suppressive myeloid cells (i.e., MDSCs). However, this suppressive phenotype can also occur in activated neutrophils.^52^ Our results point to factors in the microenvironment of advanced EOC inducing a suppressor phenotype in mature neutrophils. Mechanisms for this suppression are complex and involve a number of neutrophil effector functions (Singel et al., *manuscript under revision*). Together with other suppressive pathways (e.g., MDSCs, tumour-associated macrophages, and regulatory T cells), neutrophil-mediated suppression of T cell responses is expected to obstruct durable anti-tumour immunity.

Finally, although our results point to a pro-tumourigenic role of neutrophils in EOC, there is growing appreciation of neutrophil heterogeneity in cancer, with distinct neutrophil populations promoting cancer control or progression. It has been known for decades that activated neutrophils can kill tumour cells. More recently, Sagiv et al.^53^ identified distinct neutrophil populations in cancer with specific functional phenotypes and plasticity to switch between phenotypes. Knowledge about mechanisms by which the tumour microenvironment induces a suppressor phenotype in neutrophils may lead to new approaches to reprogram suppressive neutrophils to an anti-tumour phenotype. Our model points to products of injury, specifically mtDNA, and the subsequent neutrophil and platelet responses, as potential prognostic biomarkers and novel therapeutic targets in patients with advanced EOC. These results identified a number of neutrophil-platelet interactions and pathways to target to slow tumour progression.

## Electronic supplementary material

Supplemental Material

## Data Availability

The data sets generated during and/or analysed during the current study are available from the corresponding author on reasonable request.
