# Supplementary material for: Mitochondrial DNA in the tumour microenvironment activates neutrophils and is associated with worse outcomes in patients with advanced epithelial ovarian cancer
Source: Br J Cancer. 2018 Dec 6;120(2):207–17. doi: 10.1038/s41416-018-0339-8 (PMC6342981; doi:10.1038/s41416-018-0339-8)
Supplement: Supplementary file 1 — Supplemental Material [file 41416_2018_339_MOESM1_ESM.docx]

**Supplementary Information**

**Methods**

*Activation of Murine Bone Marrow-Derived Dendritic Cells*

BMDCs were labeled with antibodies targeted against mouse MHC Class I (#114608), MHC Class II (#107606), CD80 (#104714), and CD86 (#105028), and the respective isotype controls (Biolegend, San Diego, CA, USA).

*Quantification of Mitochondrial DNA and Genomic DNA*

We generated a standard curve using the purified liver mtDNA concentration (starting at 350 ng and followed by 10-fold serial dilutions) and used the corresponding Ct values to quantify the mtDNA concentrations found in patient ascites. Similar methods were applied to measure gDNA in patient ascites by qPCR using ascites-derived total DNA, gDNA from human liver, and human β-actin primers (forward 5’-AGAGCTACGAGCTGCCTGAC-3’ and reverse 5’-AGCACTGTGTTGGCGTACAG-3’). We used 200 μl of ascites supernatants for the DNA extraction, which was eluted into 50 μl volumes. To compensate for a concentration effect, we calculated mtDNA and gDNA concentrations (ng/μl of ascites) by dividing the Ct values by 4. All samples were analyzed in duplicate or triplicate wells and the average concentration per sample was used. Functional activity of liver-derived mtDNA was confirmed by evaluating Ca^2+^ responses in neutrophils with mtDAMPs and mtDNA *in vitro*.

*Detection of Neutrophil Extracellular Traps ex vivo*

For visualization of murine NETs, primary antibodies against mouse NE (1:100; #21595, Abcam, Cambridge, MA, USA) and histone H1 (1:100; #8030, Santa Cruz Biotechnology, Santa Cruz, CA, USA) were applied overnight at 4°C. Primary antibodies were detected with Alexa Fluor 647- and 546-conjugated secondary antibodies (1:300; #A-51447, #A-10036, Thermo Fisher Scientific, Waltham, MA, USA), respectively. For visualization of human NETs, primary antibodies against human NE (1:100; #481001, MilliporeSigma, St. Louis, MO, USA) and histone H1 were applied overnight at 4°C. Primary antibodies were detected with Alexa Fluor 488- and 546-conjugated secondary antibodies (1:300; #A-21206, #A-10036, Thermo Fisher Scientific), respectively. Slides and cover slips were fixed with mounting media with DAPI (#H-1200, Vector Laboratories, Burlingame, CA, USA). Fluorescent images were obtained from a TCS SP2 AOBS spectral confocal scanner mounted on a Leica DM IRE2 inverted fluorescent microscope using a 63X oil immersion objective lens as previously described ^33^. Intact neutrophils were identified based on cytoplasmic localization of NE (FITC) and nuclear localization of H1 (TRITC) and DAPI. NETs were identified based on the characteristic chromatin stretches with co-localized NE.

*Identification of Platelet Microparticles by Flow Cytometry*

To generate positive and negative controls for platelet microparticles (PMPs), donor peripheral blood was collected in red-top (clot-activating) and yellow-top (anti-coagulant, ACD additive) tubes, respectively. Blood in the red-top tube was allowed to clot for 20 minutes and then centrifuged at 500g for 5 minutes. Serum was collected and centrifuged at 2000g for 10 minutes. Supernatants were collected and used as a positive control for PMPs. Blood from the yellow-top tube was mixed with 6 mL of Tyrode’s buffer and centrifuged at 500g for 10 minutes. Supernatant was added to 2 mL of ACD buffer and centrifuged at 500g for 5 minutes. Supernatant was collected and centrifuged at 2000g for 10 minutes. 2000g supernatant was collected and used as a negative control for PMPs.

To visualize PMPs on the flow cytometer, we attached a sheath fluid reservoir that was connected to a 0.1 µm filter to a BD Fortessa (Becton, Dickinson and Company, Franklin Lakes, NJ, USA) flow cytometer. This allowed only for 0.1 µm sheath fluid to run through the cytometer. The sheath reservoir was allowed to pressurize and the flow rate was turned to low. In order to decrease background we also removed the drip collector from the sample aspirator. The flow rate and laser delays were recalculated. Since samples contained only one fluorescent marker, we removed all of the unnecessary filters. In filter spot A, we placed the 575/26 filter, in spot B we placed the 530/30 filter, and in spot C we placed the 488/10 filter. Calibration beads containing unlabeled and FITC labeled particles (#1493, Apogee Flow Systems, Hemel Hempstead, Hertfordshire, UK) were used to gate on particles ranging from 500 to 1000 nm. PMPs were detected based on particles that fell within the size gate (500 nm-1000 nm) and were positive for CD41a. To ensure that we were measuring platelet particles and not debris, we ran parallel samples that were 0.1 µm-filtered.

*Activation of Platelets by Ascites in vitro*

Anticoagulated blood was diluted with 1/3 volume of Tyrode’s buffer (134 mM NaCl, 2.9 mM KCl, 0.34 mM Na2HPO4, 1 mM MgCl2, 10 mM HEPES, 5 mM D-glucose, 0.3% bovine serum albumin, pH 7.4) and centrifuged at 600g for 3 min. The upper 2/3 of platelet-rich plasma was removed by aspiration and contaminating red blood cells were pelleted at 400g for 2 min followed by pelleting platelets at 1700g for 10 min. The platelets were resuspended in Tyrode’s buffer. Ascites samples were centrifuged at 500g prior to platelet stimulation. Platelets in Tyrode’s buffer were exposed to 50% volume of ascites or vehicle (Tyrode’s buffer) in the presence of 1mM CaCl_2_ and incubated at 37°C for 30 or 15 min for human or mouse platelets, respectively. During preliminary experiments, these incubation times were found to result in a close to maximal P-selectin expression, whereas no significant effect of CaCl_2_ was found on platelet P-selectin and CD42d levels up to 1h post-administration. In separate studies, ascites samples were pre-incubated with DNase I (0.05% w/v, #07900, Stemcell Technologies, Cambridge, MA, USA) and/or protease inhibitor cocktail (1:100, #539131, Protease Inhibitor Cocktail Set I, MilliporeSigma) for 30 min prior to platelet stimulation as described above. 15 μm polystyrene microbeads (#18328-5, Polysciences, Warrington, PA, USA) were also added to the labeling cocktail to obtain total cell counts and validate percentage values.

*Detection of Histones by Western Blot*

Ascites protein concentration was determined by BCA assay. All subsequent fractionation steps were performed at 4°C. One 320 µl aliquot of unfractionated ascites was kept on ice while the remaining 680 µl was centrifuged at 10 000g for 30 min. The 10 000g pellet and 320 µl of the 10 000g supernatant were retained. A fraction of all samples were taken for western blotting or platelet aggregation analysis.

Proteins were separated by SDS-PAGE on 4-15 % (w/v) Tris-Glycine gels and transferred to nitrocellulose membrane. Blots were blocked in Odyssey blocking buffer (PBS) and then incubated with anti-H3-CT antibody (1:5000; #07-690, MilliporeSigma; detects both H3 and H1), and IRDye 800CW goat anti-rabbit conjugated secondary antibody (1:8000; #926-32211, LI-COR Biosciences, Lincoln, NE, USA). The detection was performed with an OdysseyCLx infrared imaging system (LI-COR Biosciences). Band intensity analysis and histones quantification were performed using Image Studio software (LI-COR Biosciences).

*Measurement of Neutrophil Elastase and Nucleosomes by ELISA*

NE levels from banked ascites supernatants were measured by ELISA (#EE1001-1, Human Elastase Kit, AssayPro, St. Charles, MO, USA). Nucleosomes from acid-extracted ascites supernatants were measured by ELISA (#11774425001, Cell Death Detection ELISA^PLUS^, MilliporeSigma).

To acid-extract histones, ascites supernatants or serum were washed with neutralization buffer (1X PBS, 10 mM PMSF, 0.02% NaN_3_) and centrifuged 5000g, 10 min, 4°C. The supernatants were removed and kept as the histone-deplete fraction, and the pellets were kept as the histone-rich fraction. After overnight treatment with 0.2 N HCl at 4°C, the histone-rich fraction was centrifuged at 5000g, 10 min, 4°C. The enrichment factor for the nucleosome analysis was calculated by dividing the absorbance of the patient sample by the absorbance of the healthy donor serum.

**Supplemental Figures and Tables**

| N | 11 |
| --- | --- |
| Age, Mean | 64.3 |
| Stage |  |
| IIIC | 73% |
| IV | 18% |
| Unstaged | 9% |
| % Grade 3 | 91% |
| Histology |  |
| Serous | 55% |
| Mixed | 36% |
| Benign (Thecoma) | 9% |
| Debulking |  |
| Optimal | 55% |
| Sub-optimal | 27% |
| Unclear | 18% |
| Residual Tumor |  |
| R0* | 9% |
| Not R0 | 91% |
| Platinum Status |  |
| Sensitive | 36% |
| Refractory/Resistant | 27% |
| Presence of Malignant Cells | 91% |
| *R0: no residual disease |  |

**Supplemental Table 1. Clinical characteristics of patients whose ascites were used in neutrophil and platelet biological assays.** Ascites supernatants were used in Figures 2 and 3, and Supplemental Figures 4-7.

**
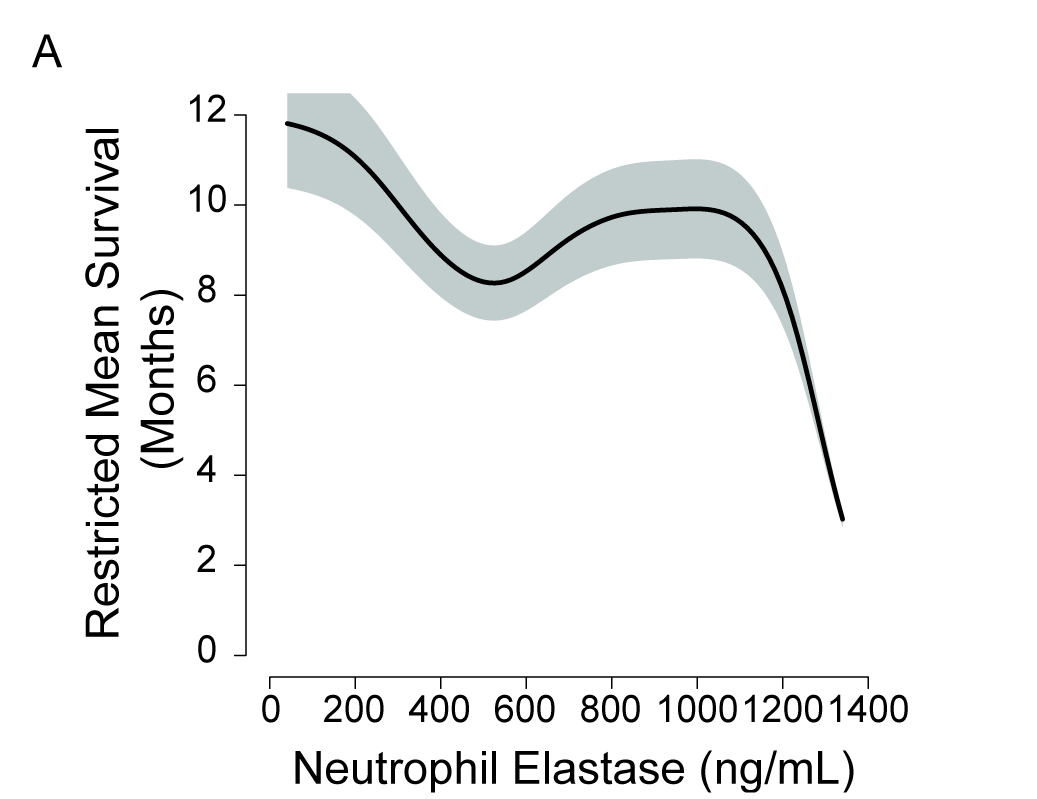
**

**Supplemental Figure 1. Neutrophil elastase (NE) in the ascites is associated with reduced progression-free survival within 12-months following surgery.** Banked ascites supernatants from patients with newly diagnosed advanced EOC were analyzed for NE (n=73). NE levels (ng/mL) were treated as a continuous variable and plotted against the restricted mean survival (RMS). At higher levels of ascites NE (e.g., 1200 ng/mL), patients had a survival of approximately 8.5 months, whereas with lower levels of ascites NE (e.g., 200 ng/mL), patients survived approximately 11.5 months.

**Supplemental Figure 2. Mitochondrial DAMPs induce generation of neutrophil extracellular traps independent of NADPH oxidase.** A) Mitochondrial DAMPs (mtDAMPs) activate murine neutrophils via NADPH oxidase (NOX2). Bone-marrow purified neutrophils (BM-PMNs) from WT and NOX2-deficient (p47^phox-/-^) mice were treated with mtDAMPs equivalent to concentrations recovered *in vivo* experimental 5% liver injury models (WT, blue line; NOX2-deficient, green line) for 15 minutes, and intracellular hydrogen peroxide was measured by flow cytometry based on DHR123 fluorescence. B) mtDAMPs induce generation of NETs independently of NOX2. BM-PMNs from NOX2-deficient mice (DNA, blue) and neutrophils (PMNs) from healthy donors (DNA, blue; NE, green) were treated with PMA (positive control for NOX2-dependent NETosis) or mtDAMPs for 1h. PMA induces NET generation dependent on NOX2 activation, whereas mtDAMPs induce NET generation independently of NOX2 activation (NETs, white arrows). Data are from ≥3 independent experiments.

**
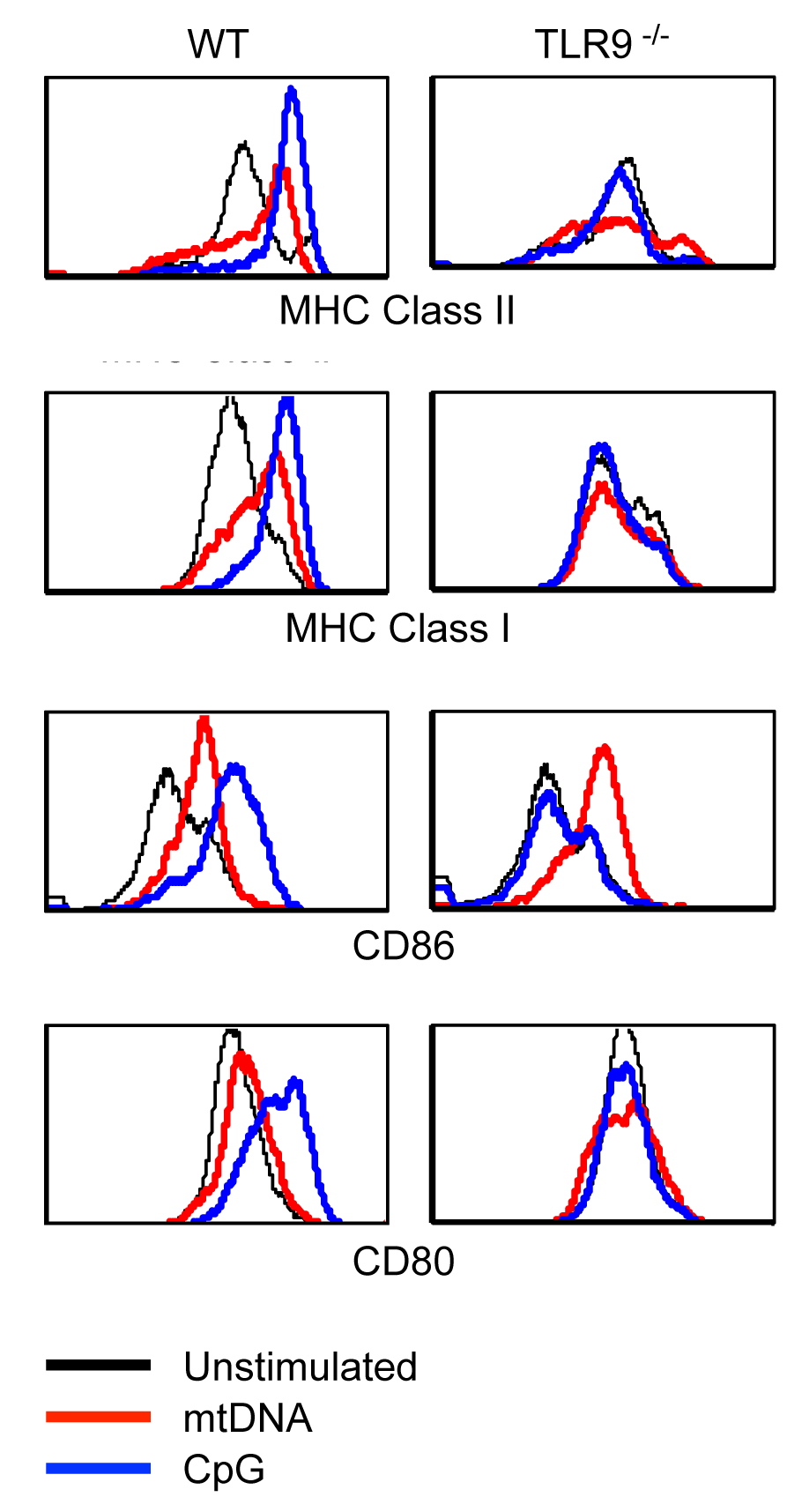
**

**Supplemental Figure 3. Mitochondrial DNA activate DCs through TLR9-dependent and -independent pathways.** BMDCs were purified from WT and TLR9-deficient mice, treated with mtDNA at clinical concentrations (1-10 µg/ml; ^32^) (red lines) or CpG sequences (positive control for TLR9-ligation; blue lines), and evaluated for surface expression of MHC class II and I, CD86, and CD80. Data are representative of ≥3 independent experiments.


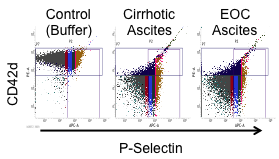


**Supplemental Figure 4. Both EOC and cirrhotic ascites induce rapid platelet activation.** Ascites were collected from patients with newly diagnosed advanced EOC and patients with liver cirrhosis; 500g supernatants were used. Naïve murine platelets were exposed to Tyrode’s buffer with 1mM CaCl_2_ (negative control), cirrhotic ascites (n=3) in the presence of 1mM CaCl_2_, or EOC ascites supernatants (n=5) in the presence of 1mM CaCl_2_ for 30 minutes prior to staining for flow cytometry. Representative density plots show increased P-selectin^+^ and loss of CD42d from the surface of platelets within 15 minutes after exposure to cirrhotic and EOC ascites supernatants.


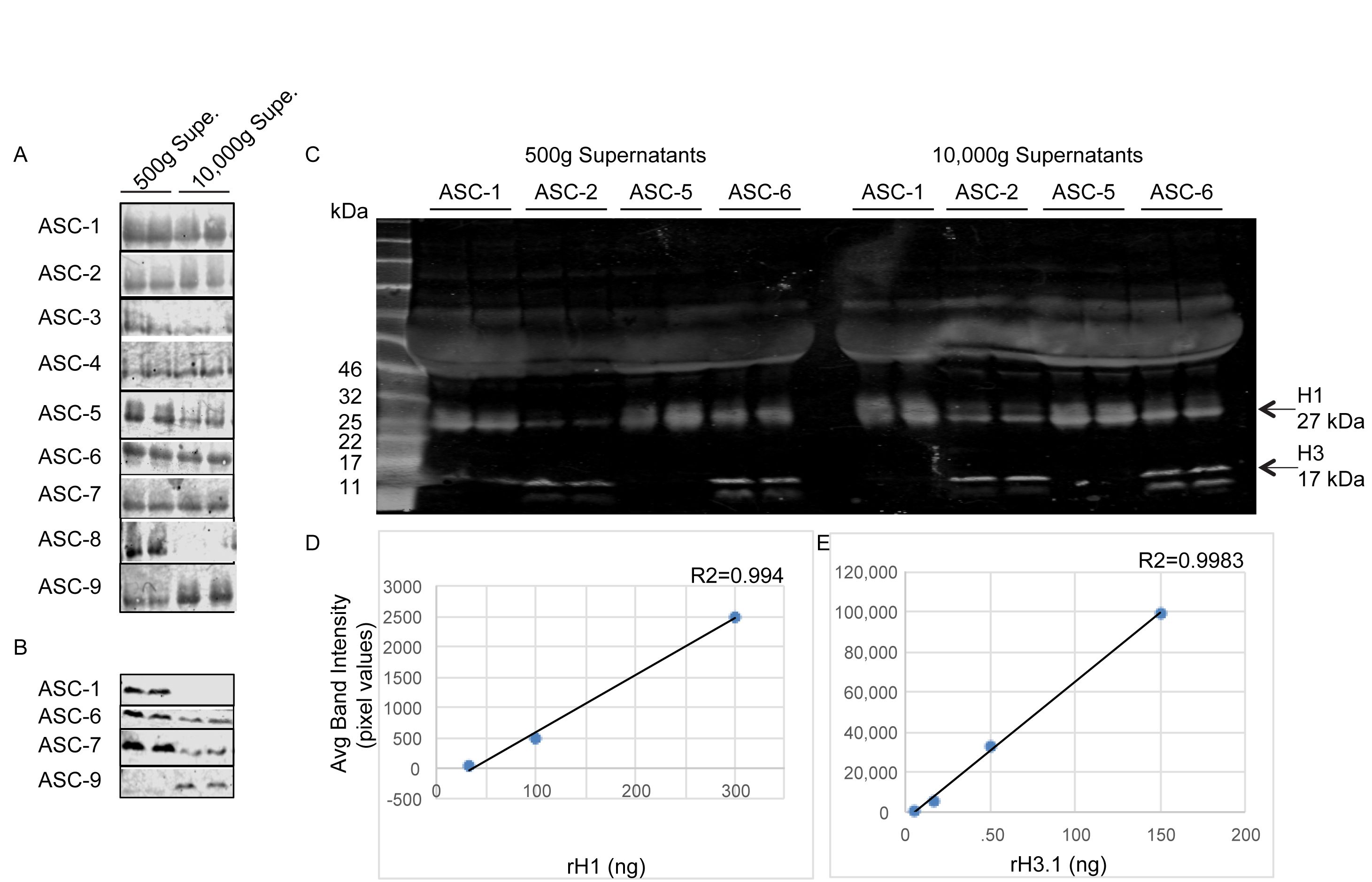


F

G


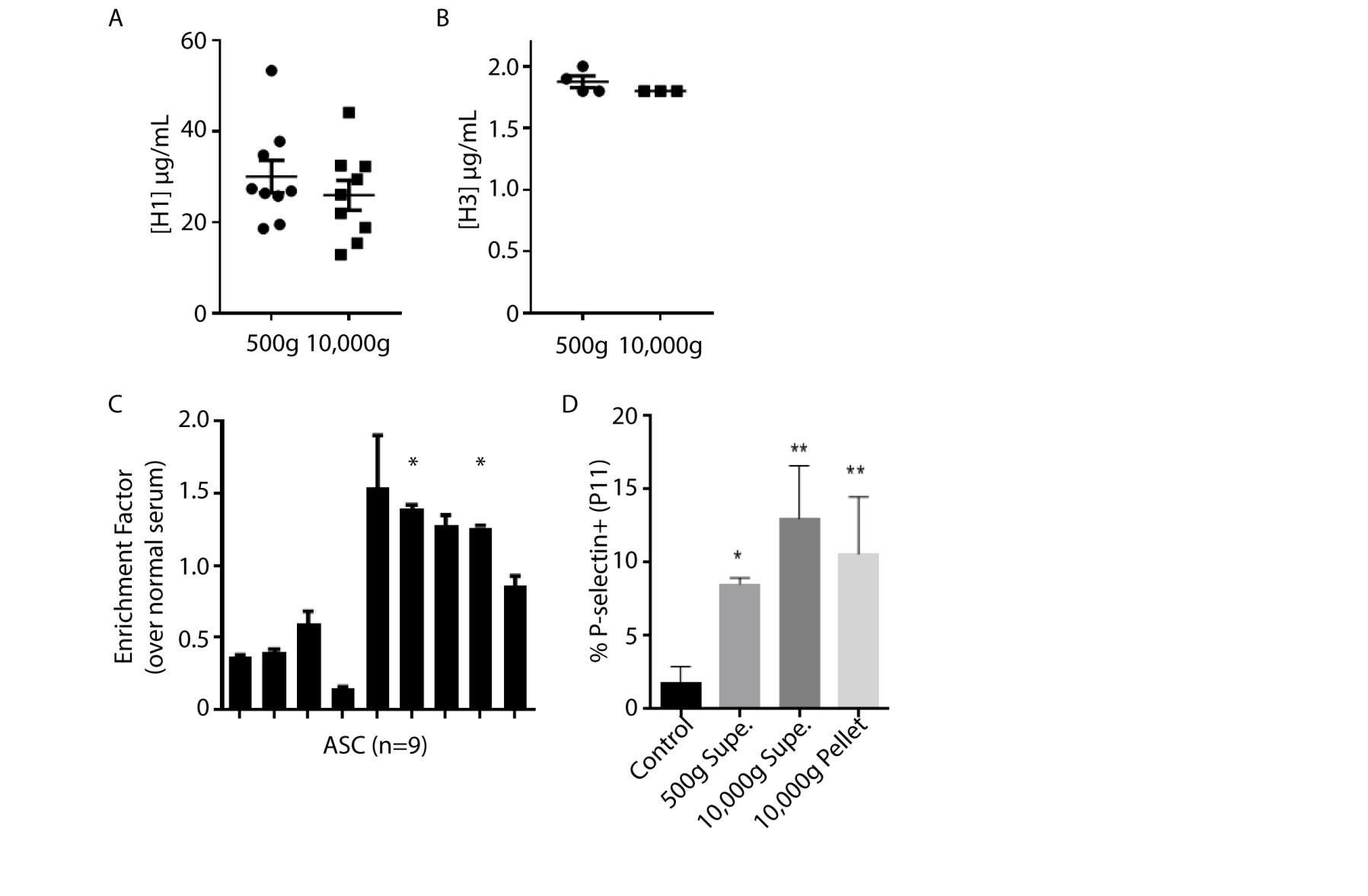

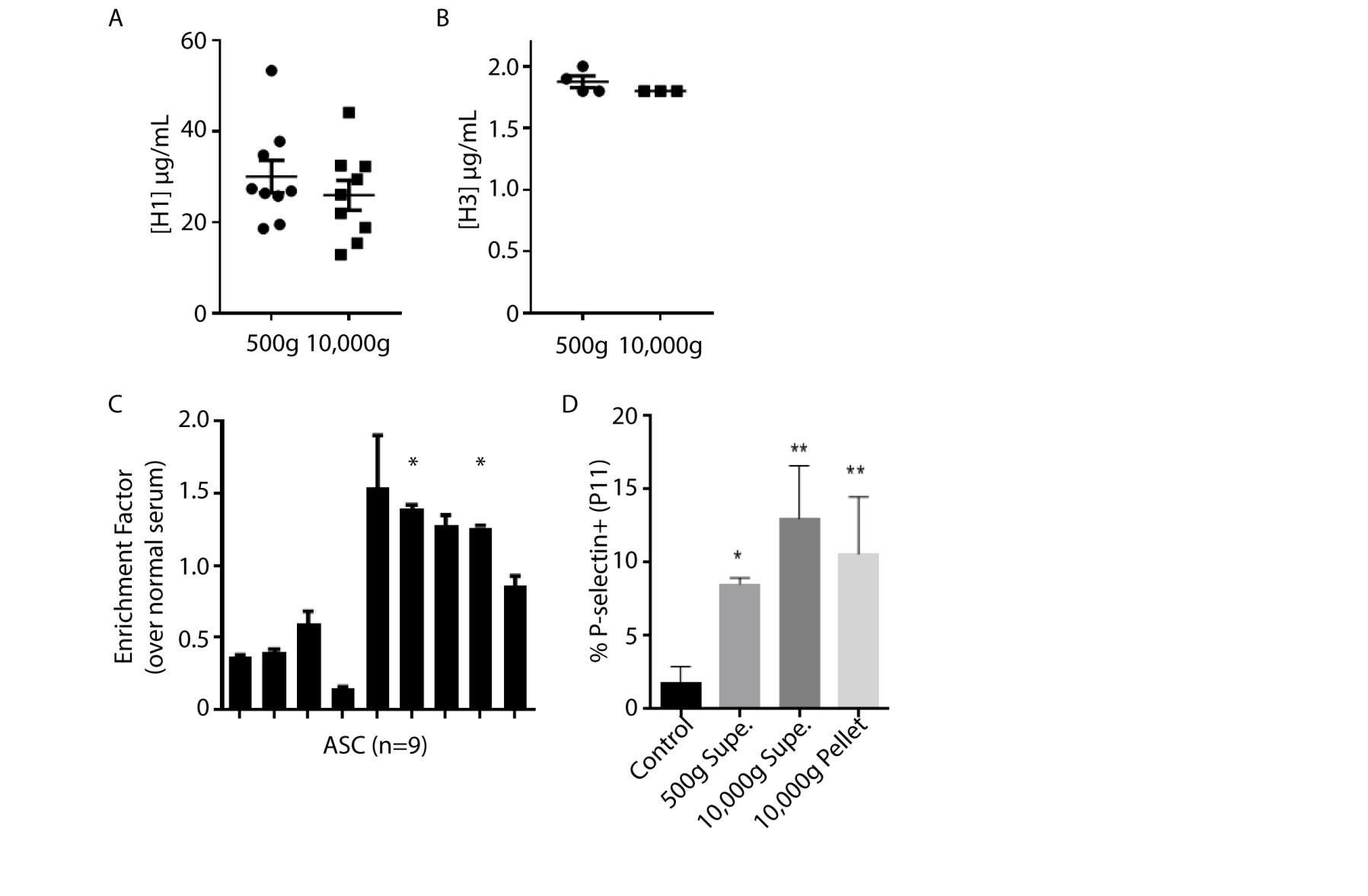


H

**Supplemental Figure 5. The concentration of histone H1 is 10-fold higher than the concentration of core histones in ascites.** Ascites were collected from patients with newly diagnosed advanced EOC and 500g supernatants (ASC) and 10 000g supernatants were used. The 10 000g centrifugation was to sediment histones associated with apoptotic bodies. A) Linker histone H1 and B) core histone H3 were quantified in ascites supernatants (n=9) by Western blot. Total protein (10 µg) was run without fractionation (500g supernatants) or the corresponding volume from the 10 000g supernatants. C) Western blot image of 500g and

10 000g ascites supernatants shows the presence of non-specific bands due to high protein concentration. Histone concentrations (µg/mL) were derived from standard curves generated with known amounts of recombinant histone D) H1 and E) H3 (kDa = kiloDalton). F) H1 and G) H3 levels were similar in 500g and 10 000g supernatants. H) Nucleosome levels were measured in acid-extracted ascites supernatants by ELISA (n=9). The enrichment factor was calculated by dividing the absorbance of the sample by the absorbance of normal serum.

Data are from ≥3 independent experiments.

**
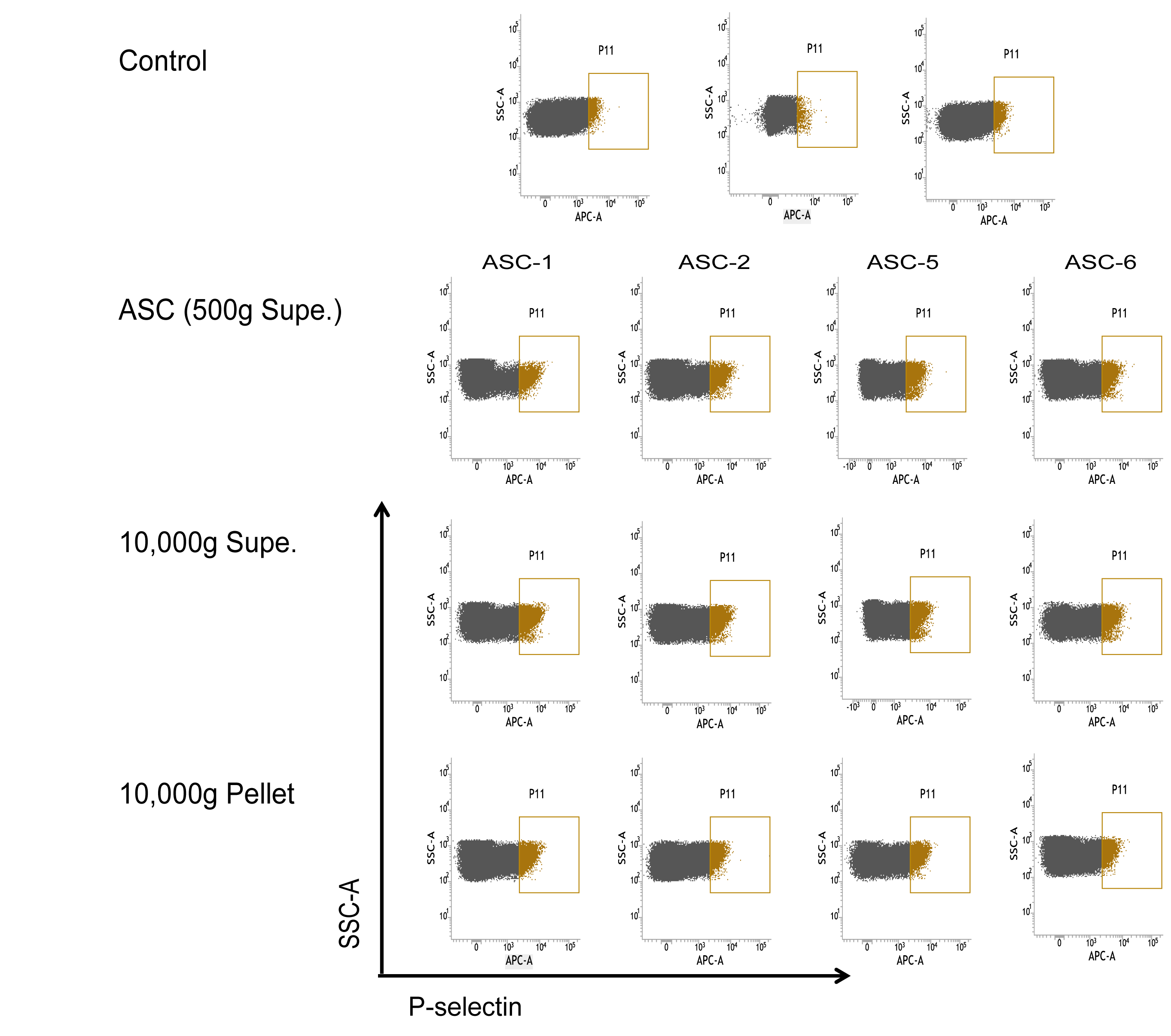
**

**Supplemental Figure 6. Incomplete histone depletion of ascites supernatants does not abrogate P-selectin up-regulation in donor platelets.** Ascites were collected from patients with newly diagnosed advanced EOC and 500g supernatants (ASC), 10 000g supernatants, and 10 000g pellets were used. The 10 000g centrifugation was to sediment histones associated with apoptotic bodies. Platelets from peripheral blood of healthy donors were exposed to Tyrode’s buffer with 1mM CaCl_2_ (negative control), ascites 500g supernatants in the presence of 1mM CaCl_2_, and 10 000g supernatants or pellets in the presence of 1mM CaCl_2_ for 30 minutes prior to evaluating for P-selectin expression by flow cytometry. Density plots are shown for individual ascites tested (n=4). Data are representative of ≥3 independent experiments.

**
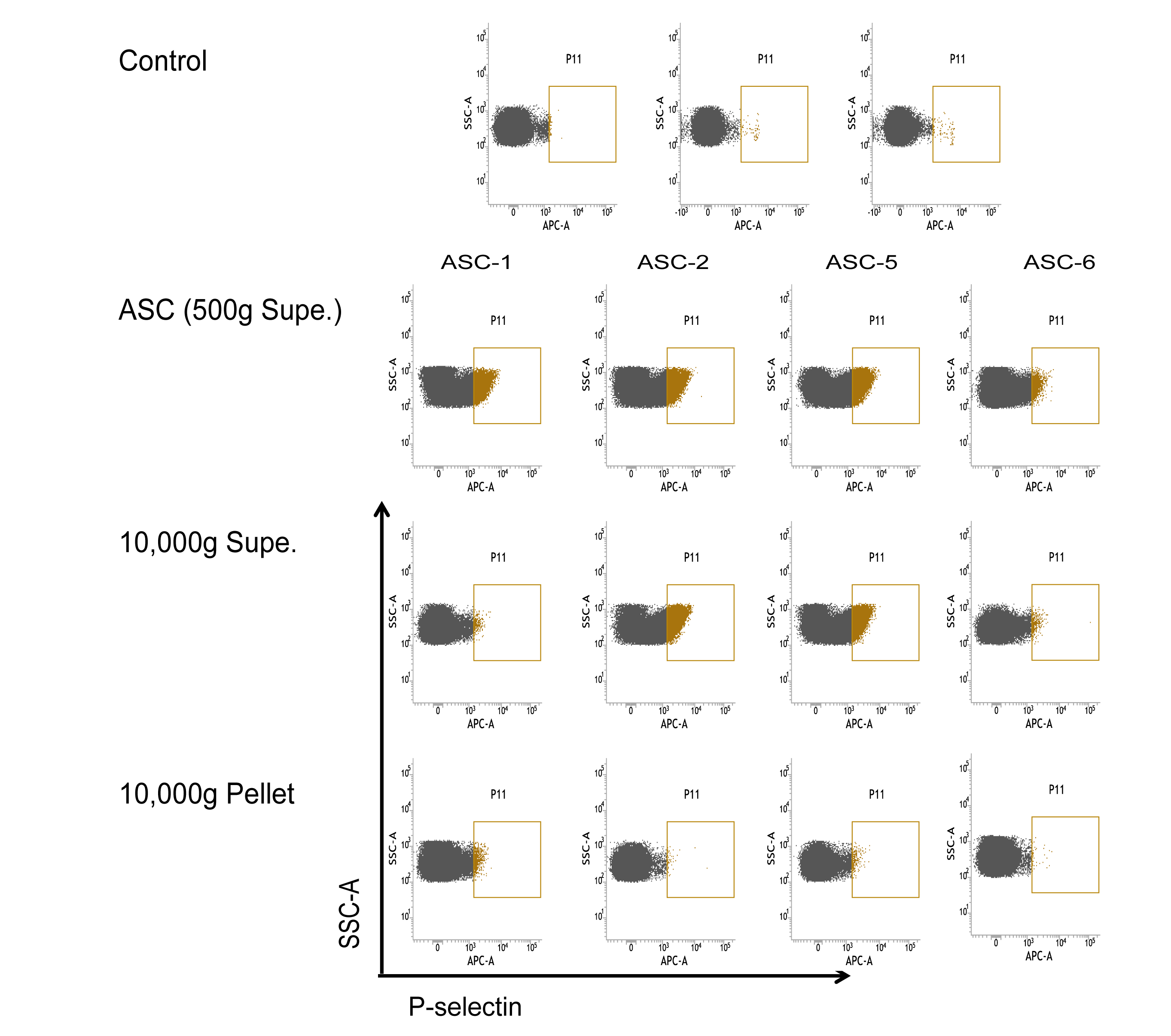
**

**Supplemental Figure 7. Incomplete histone depletion of ascites supernatants variably abrogates P-selectin up-regulation in murine platelets.** Ascites were collected from patients with newly diagnosed advanced EOC and 500g supernatants (ASC), 10 000g supernatants, and 10 000g pellets were used. The 10 000g centrifugation was to sediment histones associated with apoptotic bodies. Murine platelets were exposed to Tyrode’s buffer with 1mM CaCl_2_ (negative control), ascites 500g supernatants in the presence of 1mM CaCl_2_, and 10 000g supernatants or pellets in the presence of 1mM CaCl_2_ for 30 minutes prior to evaluating for P-selectin expression by flow cytometry. Density plots are shown for individual ascites tested (n=4). Data are representative of ≥3 independent experiments.
